# Divine Providence

**Published:** 1888-11

**Authors:** 


### DIVINE PROVIDENCE.

We learn that among the late victims of the yellow fever epidemic that almost emptied Jacksonville of its population, was a bravely benevolent physician of Louisville, who, leaving home and family and friends behind, threw himself into the midst of the sick and the dying in the endeavor to restore them to health. After a few days of incessant attention upon the sufferers, this noble volunteer was stricken with the fever and died. Then it was that a number of his surviving friends met and passed complimentary resolutions to be laid upon his grave, beginning with the hackneyed prelude "Whereas it has pleased Divine Providence to remove from our midst," etc.

Let us pause right here and critically examine this sentiment in what might be said to be a doctrinal aspect. That the yellow fever proceeds from purely natural or God-given causes will be conceded. The prevailing opinion among those who should know best, is that it is the outgrowth of certain *microbes*, sometimes called *bacteria*, that swarm in the air by millions on millions, against which there is no known defense, the neighborhood result being disease and death to the human race. Jacksonville, formerly so prosperous and abounding in happy homes, furnishes a melancholy example of the inevitable law.

In the light of these resolutions it must be assumed that it pleased God to visit this terrible scourge upon its indwellers.

One of the most lauded elements of a true manhood is charity—charity to the suffering and the desolate, charity to the living and the dead, and he who was so noble as to take his life in his hand and risk his all that others might survive the deadly pestilence, furnishes, according to popular view, a sublime example of the Christ spirit in man.

Why, then, in the language of the before-mentioned resolutions, did it "please God to remove him," to overcome with death this instrument of pure benevolence in the hour of his greatest need? Is it because He was displeased with his endeavor to stay the ravages of the pestilence? Does not the one view logically infer the other? Is it not about time we should be done with this sort of hypocritical cant and find some more worthy solution of the common events pertaining to this life?

